# Influence of cell seeding density and inoculation timing on HepG2 cell engraftment on the chick chorioallantoic membrane

**DOI:** 10.1016/j.mex.2026.104241

**Published:** 2026-11-03

**Authors:** Cristina Adriana Dehelean, Diana Haj Ali, Robert Barna, Iasmina Predescu, Ioana Macasoi

**Affiliations:** a Faculty of Pharmacy, "Victor Babeș" University of Medicine and Pharmacy Timișoara, 2 Eftimie Murgu Square, 300041, Timișoara, Romania; b Research Center for Pharmaco-Toxicological Evaluations (FARMATOX), "Victor Babeș" University of Medicine and Pharmacy Timișoara, 2 Eftimie Murgu Square, 300041, Timișoara, Romania; c Faculty of Medicine, "Victor Babeș" University of Medicine and Pharmacy Timișoara, 2 Eftimie Murgu Square, 300041, Timișoara, Romania; d ANAPATMOL Research Center, Faculty of Medicine, "Victor Babeș" University of Medicine and Pharmacy Timișoara, 2 Eftimie Murgu Square, 300041, Timișoara, Romania

**Keywords:** Chorioallantoic membrane, In ovo xenograft model, HepG2 cells, Cell density, Grafting day, Histology, Immunohistochemistry

## Abstract

Preclinical research on liver cancer requires models that closely mimic the biological environment, including three-dimensional tumor growth and vascularization, while ensuring rapid, inexpensive, and ethically favorable experimental conditions. Against this background, the chick embryo chorioallantoic membrane (CAM) offers a highly vascularized, immunologically immature (not yet immunocompetent) in ovo environment favorable for the engraftment of human tumor cells and in accordance with the 3R principles. HepG2 is the most widely used human hepatic tumor cell line, and although it is recognized as a malignant hepatic tumor, its precise classification remains debated. In this article, two cell densities (0.5 × 10⁶ and 1 × 10⁶ cells) were compared at three engraftment times (embryonic day 7, 8, and 9) to define reproducible engraftment conditions. Cohesive, histologically processable grafts were obtained only at the highest cell density, grafted on day 9, with a success rate of 85% (17/20 eggs). The five remaining conditions yielded no processable graft (0/100). Hematoxylin-eosin staining showed a cellular mass on the membrane, whereas the immunohistochemical panel (HepPar-1, Arginase-1, pan-cytokeratin) showed focal, heterogeneous staining. Therefore, the findings are compatible with a malignant hepatocellular tumor. The protocol is reported together with the five unsuccessful conditions as a practical reference for laboratories setting up the assay.

• Cell density and grafting day determine cohesive HepG2 graft formation on the CAM.
• Grafts formed only with 1 × 10⁶ cells on embryonic day 9 (17/20); others 0/100.
• Five suboptimal conditions are reported as a CAM assay troubleshooting reference.

## Graphical abstract

**Figure:**
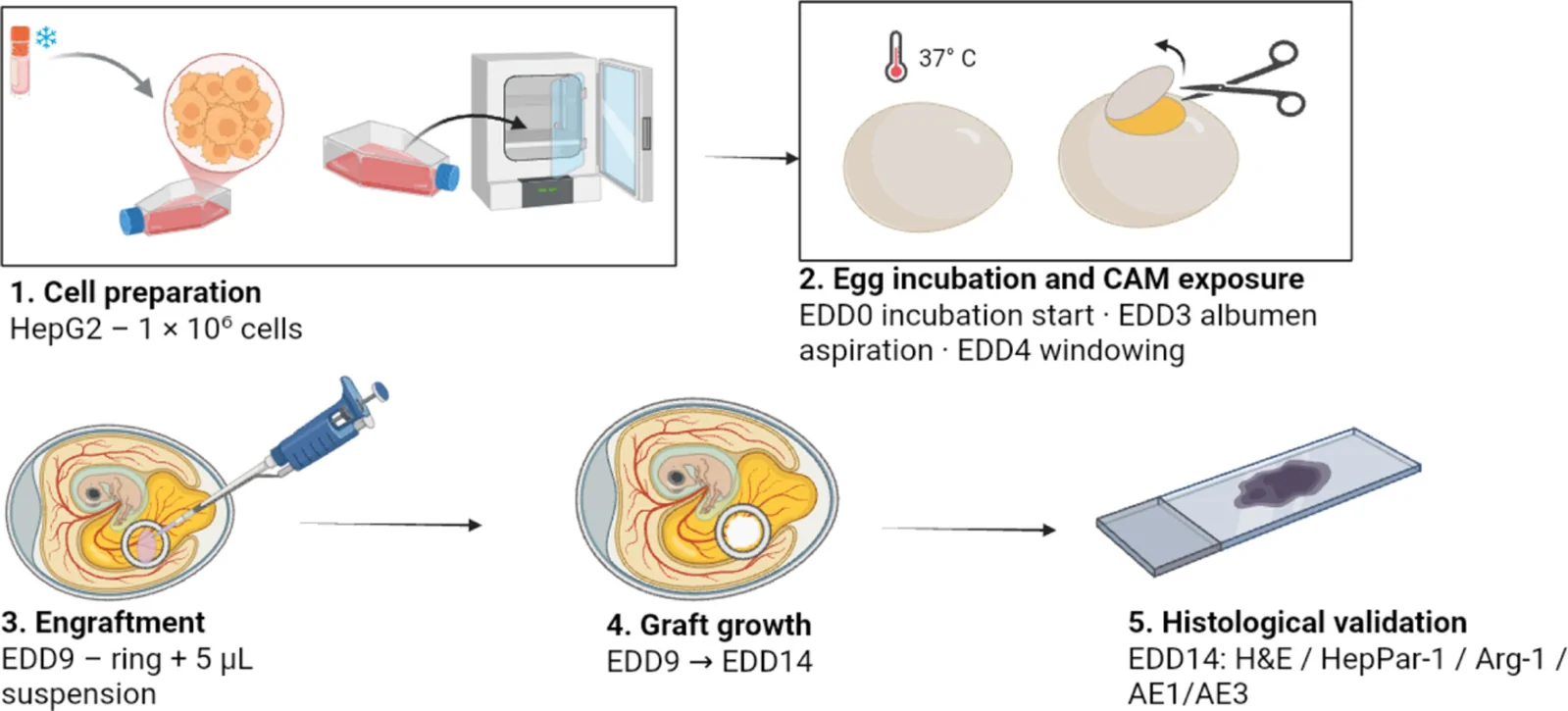

**Specifications table:**

**Table:** 

Subject area	Biochemistry, Genetics and Molecular Biology
**More specific subject area**	In ovo tumor modeling; effect of cell seeding density and grafting day on HepG2 cell engraftment on the chick chorioallantoic membrane (CAM)
**Name of your method**	Optimized method for engraftment of HepG2 cells onto the chorioallantoic membrane (CAM)
**Name and reference of original method**	1. Li M, Pathak RR, Lopez-Rivera E, Friedman SL, Aguirre-GhisoJA, Sikora AG. The in ovo chick chorioallantoic membrane (CAM) assay as an efficient xenograft model of hepatocellular carcinoma. J Vis Exp. 2015;(104):e52411. https://doi.org/10.3791/52411 2. Avram S, Coricovac DE, Pavel IZ, Pinzaru I, Ghiulai R, Baderca F, et al. Standardization of A375 human melanoma models on chicken embryo chorioallantoic membrane and Balb/c nude mice. Oncol Rep. 2017;38(1):89–99. https://doi.org/10.3892/or.2017.5658
**Resource availability**	***Reagents*** HepG2 cell line, used as a human hepatic tumor model (ATCC, Cat. No HB-8065; RRID:CVCL_0027) EMEM culture medium (ATCC, Cat. No 30–2003) Fetal bovine serum, FBS (ATCC, Cat. No 30–2020) Penicillin (100 U/mL)–Streptomycin (100 μg/mL) (PAN-Biotech, Cat. No P06–07,100) Trypsin 0.25% / EDTA 0.02% in PBS, w/o Ca and Mg, with phenol red (PAN-Biotech, Cat. No P10–019,500) PBS, Phosphate Buffered Saline (Sigma, Cat. No D1408–500ML) Trypan Blue (Sigma, Cat. No T8154) Hematoxylin (Richard-Allan Scientific, Catalog no 7211) Eosin Y (Richard-Allan Scientific, Catalog no 7111) Clarifier 1 (Richard Allan Scientific, Catalog no 7401) Bluing Reagent (Richard-Allan Scientific, Catalog no 7301) BOND Epitope Retrieval Solution 2 (Catalog no.: AR9640) Enzyme Pretreatment Kit (Catalog no.: AR9551) Bond Polymer Refine Detection (Catalog no.: DS9800) FLEX Anti-Human Hepatocyte (Agilent; Clone: OCH1E5, Ready-to-Use, Mouse Monoclonal; Cat. No.: GA624) Multi-Cytokeratin (Leica Biosystems; Clone: AE1/AE3, Ready-to-Use, Mouse Monoclonal; Cat. No.: PA0909) Arginase-1 (Diagnostic BioSystems; Clone: DBM15.12; Ready-to-Use, Mouse Monoclonal, Cat. No.: PDM197). ***Supplies*** T75 culture flask (Eppendorf, Cat. No 00–30,710,126) 15 mL Falcon tube (Eppendorf, Cat. No 30,122,151) 1.5 mL Eppendorf tube (Eppendorf, Cat. No 30,120,086) 10% neutral buffered formalin (Epredia, Cat. No 5735-LP) Histological sponge (CellPath Ltd., Cat. No EBA–0106–03A) Histology cassette (VWR-Avantor, Cat. No QPATH 720–2215) Paraffin, triple-filtered, melting point 56–57 °C (Epredia, Histoplast PE 8330) Silk adhesive tape (OctaCare) Positive charge slides for immunohistochemistry (Deltalab, Cat. No D100018) Mounting Medium (Histocore Spectra CV X; Leica Biosystem Richmond Inc Producator, Cat. No 3801,733) ***Equipment*** Digital Bird Incubator MX-50D (Rcom Incubator USA) Centrifuge 5810R (Eppendorf) Bright-Line Hemacytometer (Hausser Scientific) EVOS XL Core microscope (Invitrogen, Thermo Fisher Scientific) Tissue processor, Leica TP1020 Paraffin embedding station, Arcadia H+C Microtome, Leica RM2245 Automated tissue stainer, Medite Tissue Stainer TCA-44 BOND-MAX Fully Automated IHC and ISH Staining System, Leica Aperio AT2 scanner, Leica Discovery V8 stereomicroscope with Axiocam 105 camera, Zeiss ***Software*** ImageJ/Fiji (version 1.53) GraphPad (version 11)

## Background

Malignant hepatocellular tumor (MHT), taken together as hepatoblastoma or hepatocarcinoma, is presently among the most prevalent primary liver malignancies and constitutes one of the primary causes of cancer-related mortality globally. The main challenge associated with this type of cancer is its frequent diagnosis at advanced stages, because early-stage tumors cause no specific symptoms and routine liver screening is often underutilized [1]. To advance the development of novel effective therapies, it is essential to establish and employ pertinent preclinical models capable of replicating three-dimensional tumor growth and the associated vascularization characteristic of the neoplastic process [2,3]. While murine models are regarded as the standard, they possess significant disadvantages including high costs, extended experimental durations, the requirement for ethical approval for animal studies, and limitations concerning the tumor-immune system interface [4,5].

The chorioallantoic membrane (CAM) of the chicken embryo represents a valuable, highly vascularized, and immunologically immature, not yet immunocompetent, in ovo alternative method, which allows for the efficient and rapid implantation and growth of human tumors [2,6]. Additionally, the model aligns with the 3Rs principles: under Directive 2010/63/EU and the corresponding national legislation, avian embryos before hatching are not considered protected animals [7,8]. All procedures in this methodology were performed up to embryonic development day 14 (EDD14; the notation EDD is used throughout this article and is equivalent to the embryonic day, ED, used in part of the CAM literature), before hatching and before the onset of nociceptive competence [9]. Therefore, it does not fall under experimental considerations regarding laboratory animals. The optimal window for grafting is between days 8 and 10, when the angiogenic receptivity of the membrane is at its maximum [2,4].

Grafting of hepatic tumor cells onto the chorioallantoic membrane (CAM) was previously described by Li and colleagues, who detailed a protocol yielding vascularized tumors with a poorly differentiated hepatocellular phenotype [10]. The grafting conditions have not, however, been systematically compared for the HepG2 line. HepG2 is the most widely used human malignant hepatic tumor cell line, but its origin has been debated between hepatocellular carcinoma and hepatoblastoma, lately being re-assigned to hepatoblastoma on the basis of cytogenetic and molecular evidence [11], and its transcriptional profile is among the least representative of resected hepatocellular carcinoma tissue [12]; it is therefore used here as a human malignant hepatic tumor model rather than as a model of hepatocellular carcinoma, consistent with the approach taken in the present study. Cell density and the day of grafting are the two parameters most likely to govern engraftment success, and they have not been assessed comparatively for HepG2 within a single standardized experimental framework [10,13].

Based on the chorioallantoic membrane (CAM) grafting protocol published by Li et al. and the standardized melanoma model previously developed by our research group, the present study establishes the optimal conditions under which HepG2 cells form a compact, homogeneous graft compatible with histological processing on CAM [10,13]. Two cell densities and three grafting times were compared, all integrated into a single and standardized methodological framework. Conditions that did not lead to the formation of viable grafts are explicitly documented, to serve as practical methodological troubleshooting points. Grafts obtained under optimal conditions were characterized by histological examination and a panel of immunohistochemical markers; this characterization is descriptive in nature and is presented in the following sections, together with the associated technical limitations.

## Method details

### Cell culture and preparation of HepG2 cell suspension

HepG2 cells (ATCC, catalog number HB-8065™) were purchased, as a frozen vial. For the experiment, the cells were cultured in the specific culture medium represented by EMEM (ATCC, catalog number 30–2003). The culture medium was supplemented with 10% fetal bovine serum (FBS, ATCC, catalog number 30–2020) and 1% penicillin (100 U/mL)–streptomycin (100 μg/mL) (PAN-Biotech, catalog number P06–07,100). The cells were grown in a controlled environment, in an incubator set at 37 °C and 5% CO₂.

At the moment of reaching an approximate 80% confluence, the cells were trypsinized. The cell line, at the time of grafting to the chorioallantoic membrane, was at passage 8. The first step involved removing the culture medium from the T75 flask (Manufacturer of the flask – Eppendorf, catalog number 00–30,710,126) and washing the cells with PBS (Phosphate Buffered Saline, Sigma, catalog number D1408–500ML). Then, 3 mL of Trypsin 0.25% / EDTA 0.02% in PBS, without calcium and magnesium, with phenol red (PAN-Biotech, catalog number P10–019,500) was added to the T75 flask, and the cells were left in contact with it for approximately 5 min at 37 °C until they detached from the flask. The trypsin was inactivated by adding 6 mL of culture medium, and the cell suspension was transferred into a 15 mL Falcon tube (Eppendorf, catalog number 30,122,151). The cells were centrifuged for 5 min at 1700 rpm (553 × g, Eppendorf A-4–81 rotor) using a Centrifuge 5810R (Eppendorf). Afterwards, the supernatant was removed, and the cell pellet was resuspended in 1 mL of culture medium. A volume of 10 μL of this cell suspension was taken and mixed with 50 μL of Trypan Blue (Sigma, catalog number T8154). From this mixture, 10 μL was placed on the counting chamber (Bright-Line Hemacytometer, Hausser Scientific, Horsham, PA, USA). The EVOS XL Core microscope (Invitrogen, Thermo Fisher Scientific) was used for cell counting. The number of cells in the suspension was determined by counting the cells in the four quadrants of the hemocytometer and applying the calculation formula: total cell count/mL = arithmetic mean of the four quadrants × 6 × 10⁴, where 6 is the dilution factor. Finally, the volume corresponding to a count of 0.5 × 10⁶ and 1 × 10⁶ cells was calculated, and this volume was transferred into a 1.5 mL Eppendorf tube (Eppendorf, catalog number 30,120,086). The tubes were centrifuged again for 5 min at 1700 rpm (553 × g). The supernatant was removed, and the cell pellet was resuspended in a final volume of 5 μL of medium. This volume represented the final volume applied to the chorioallantoic membrane. Cell viability was assessed immediately before engraftment by the trypan blue exclusion method and was at least 85% in all experiments. The interval between final resuspension of the cell pellet and application of the suspension to the CAM did not exceed 5 min.

### Egg incubation and CAM exposure

Fertilized chicken eggs (gallus domesticus; mixed-breed layer flock, genetic line not defined; 52–65 g) were sourced from a small local family farm in Timis County, Romania. This farm is not a registered commercial hatchery, and the eggs were not specific-pathogen-free. The producer checked fertilization by candling at collection, and we confirmed it in our laboratory as described below. All eggs used in the study were from a single purchase, stored at room temperature for up to three days before use, and cleaned with a dry cloth without chemical disinfection. We recorded the incubation start date on each egg shell.

Eggs were placed horizontally and incubated at the same time in three Digital Bird Incubator MX-50D units (Rcom, Rcom Incubator USA) set to 37 °C and 65% relative humidity. Each incubator represented one of the three replicate runs. Using a single batch of eggs across all three incubators helped avoid variation from pre-incubation storage time, collection temperature, and flock condition, so comparisons between experimental conditions were not affected by differences between egg batches. The day on which the eggs were placed in the incubator was designated embryonic development day 0 (EDD0); all subsequent time points are given as EDD followed by the number of completed incubation days. On EDD3, albumen was aspirated from each egg through two small perforations with distinct functions. The first, made at the pointed end of the egg, was used to withdraw approximately 5–7 mL of albumen with a 10 mL syringe. The second, made at the blunt end over the natural air chamber, acted as an air inlet: it prevents the negative pressure that would otherwise develop inside the sealed shell during aspiration and allows the withdrawn albumen to be replaced by air, so that the chorioallantoic membrane detaches (‘drops’) from the inner shell membrane and an artificial air chamber is formed beneath the future window [13]. This detachment is what allows the window to be cut on EDD4 without injuring the membrane. The volume aspirated lies at the upper end of the range used in published in ovo CAM protocols; it was selected because, with eggs incubated horizontally and the window cut on the following day, a larger artificial air space produced a more reproducible detachment of the CAM from the inner shell membrane and reduced accidental injury to the membrane during windowing. Embryonic survival was not compromised at this volume: 83.3% of grafted embryos (100 of 120) were alive at EDD14, with no difference between experimental conditions (Supplementary Table S1). Laboratories that cut the window on the same day as aspiration will normally obtain adequate CAM dropping with 3–5 mL, and we recommend using the smallest volume that reliably detaches the membrane. Afterwards, the two openings were covered with silk adhesive tape (OctaCare). On EDD4, a window was made in the upper part of the eggshell by cutting it, allowing visualization of the chorioallantoic membrane. The window was covered with silk adhesive tape, and the eggs were reintroduced into the incubator until the start of the experiment.

### Grafting onto the chorioallantoic membrane: cell density and timing

The grafting of tumor cells was performed at three time points - EDD7, EDD8, and EDD9, using two cell densities: 0.5 × 10⁶ and 1 × 10⁶ cells. These two cell densities were selected for three primary reasons. First, they encompass the range most frequently applied to epithelial tumor cells in the in ovo CAM model, where 1 × 10⁶ cells per egg is a commonly used value [10,13]. Second, these densities are compatible with the matrix-free format employed in this study, in which cells are delivered in 5 µL within a 5 mm ring. At densities of 2 × 10⁶ cells and above, the suspension cannot be applied reproducibly within this volume, and published protocols using such densities deliver them in a substantially larger volume together with a matrix. Third, the aim was to identify the lowest density that reliably yields a processable graft, rather than the highest tolerated density, to optimize cost and align with the 3Rs principles. For each combination of grafting day and cell density, 20 eggs were used across three replicate runs. Each run was conducted in a separate incubator using eggs from the same batch to assess procedural reproducibility. In total, 160 eggs were set for incubation. Fertility and embryo viability were verified by candling on EDD3, before albumen aspiration, and again immediately before grafting. On EDD3, 147 eggs (91.9%) contained a viable embryo, and 140 (87.5%) were successfully windowed on EDD4. Of these, 120 eggs carrying a living embryo with an intact, well-vascularised CAM were allocated to the six experimental conditions, with 20 eggs per condition. These were distributed across the three replicate runs as 7, 7, and 6 eggs per condition, corresponding to 42, 42, and 36 grafted eggs per run. The remaining 20 windowed eggs were retained as reserves and were not grafted. The complete flow of eggs through the study, including embryo survival per condition, is summarized in Supplementary Table S1. On the day of cell grafting, a polyethylene ring was applied to the chorioallantoic membrane. The ring was made in the laboratory by cutting a Pasteur pipette made of polyethylene with a microtome blade. The result was a ring approximately 5 mm in diameter and about 1 mm thick. Inside the ring, the chorioallantoic ectoderm was superficially lacerated with the tip of a sterile 1–10 µL pipette tip. This step is necessary because the ectoderm is a one- to two-cell-thick epithelial layer that separates material deposited on the membrane from the underlying mesoderm and its capillary plexus; its partial disruption exposes the capillary bed and elicits a local wound-healing and angiogenic response, which together promote attachment and vascularisation of the graft. The laceration was restricted to the surface epithelium within the ring, macroscopically visible vessels were avoided, and no bleeding was induced, since hemorrhage inside the ring interferes both with engraftment and with the subsequent macroscopic evaluation of the graft. Finally, 5 µL of cell suspension prepared in culture medium, without any added extracellular matrix, was applied inside the ring. The matrix-free format was chosen deliberately. Matrigel is an undefined basement membrane extract derived from the Engelbreth-Holm-Swarm murine sarcoma, whose protein composition and endogenous growth factor content are not fully defined, and it would therefore introduce an uncontrolled pro-angiogenic and pro-proliferative variable into a model whose principal readouts are engraftment and vascularisation. Matrix-free grafting in a small volume has been applied successfully in the in ovo CAM model, including in the protocol previously standardized by our group [13]. After applying the cell suspension, the eggshell window was covered with adhesive tape, and the eggs were returned to the incubator under the same controlled temperature and humidity conditions (37 °C, 65% relative humidity) until day EDD14. All grafts were observed until the same day of embryonation, on day EDD14, resulting in growth durations of 7, 6, and 5 days for the grafts performed at EDD7, EDD8, and EDD9, respectively.

Graft formation was assessed in two steps, of which the second was decisive. At EDD14, a graft was first considered macroscopically present when a newly formed, opaque mass was visible within the ring, could be excised together with the underlying membrane without fragmenting, and retained its structural integrity throughout fixation, dissection and paraffin embedding, yielding sections suitable for histological evaluation. A graft was then classified as confirmed only when haematoxylin–eosin sections demonstrated a cellular mass composed of a proliferation of atypical polygonal cells in a solid and focally trabecular arrangement, with nuclear atypia and mitotic figures, and therefore distinguishable from inflammatory cells, fibrin deposition accumulation, inflammatory exudate fibrous tissue healing or chorioallantoic membrane tissue alone. Material visible within the ring that disintegrated at any step, or that did not meet the histological criterion, was recorded as an unconfirmed graft. Because a graft that fragments during harvesting is counted as unconfirmed irrespective of its histological nature, the criterion is conservative and can only underestimate the engraftment rate. An independent assessment by a second observer was not performed.

### Post-engraftment incubation and harvesting

On EDD14, the eggs were placed in a freezer at −20 °C for 5 h to induce hypothermia and euthanise the embryo. This method was selected because the protocol requires fixation in situ: the egg remains closed and undisturbed, and the fixative is subsequently introduced over the chorioallantoic membrane through the existing window, so that the graft and its interface with the membrane are preserved in their original spatial relationship. Decapitation, although faster, requires access to the embryo, which at EDD14 lies beneath the chorioallantoic membrane, and would disrupt the graft before any fixative had been applied. EDD14 corresponds to 67% of the 21-day incubation period, below the 80% threshold up to which cooling and freezing are accepted for avian embryos [14]. Laboratories that wish to apply a more conservative welfare standard may replace hypothermia with an anesthetic overdose administered through a chorioallantoic vessel before fixation, which is compatible with the present protocol and preferable on welfare grounds [9]. Then, 10% neutral buffered formalin was applied over the chorioallantoic membrane until it filled the egg up to the upper margin of the shell window used for CAM access, and the eggs were left overnight (but no >24 h) at room temperature for in situ fixation. The following day, the chorioallantoic membrane was incised with scissors to dissect the tumor together with at least a 2 mm margin of non-tumoral surrounding membrane. The collected fragment was placed on a histological sponge (CellPath, Catalog no EBA-0106–03A) within a histology cassette (QPATH 720–2215) as seen in Fig. 1. To preserve the tissue until the start of histological processing, the cassette was immersed in 10% neutral buffered formalin, ten times the sample volume, for no >24 h. Therefore, the total fixation time (in situ fixation before dissection plus immersion after dissection, until histological processing) was up to 48 h and did not exceed this, which is within the range generally accepted for immunohistochemistry on FFPE tissue, including small biopsies. For specimens of this size, the post-dissection immersion step may be shortened, with the tissue processed immediately after dissection, so that the total fixation time does not exceed approximately 24 h. The hypothermia euthanasia step preceded chemical fixation of the samples. The possible impact of this procedure on tissue morphology and antigen preservation is discussed in the limitations section of the study.

**Fig. 1:**
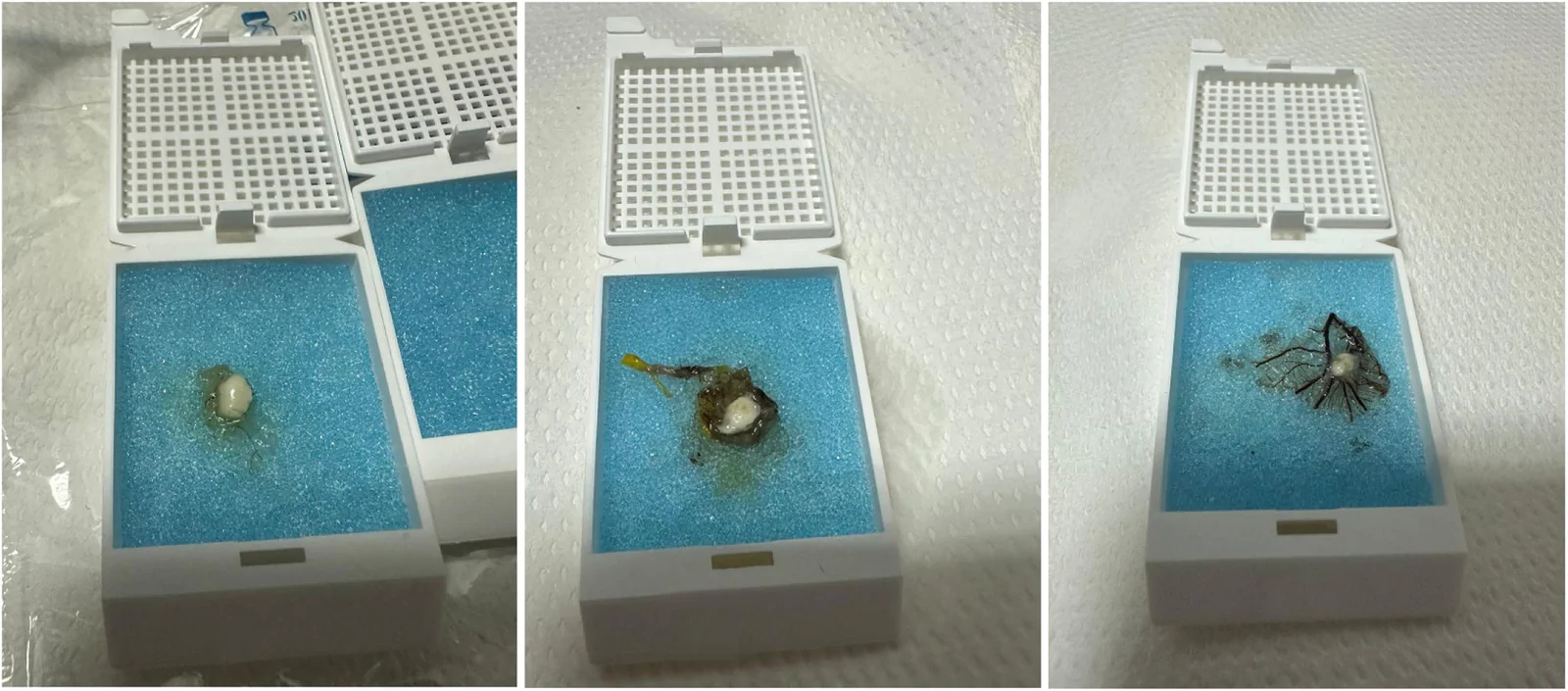
Representative images of three HepG2-derived tumors engrafted on the chorioallantoic membrane. Following dissection with a margin of surrounding non-tumoral membrane, the specimens were placed on a histological sponge within a histology cassette in preparation for tissue processing.

Tissue processing was performed using a Leica TP1020 tissue processor, following the processing protocol detailed in Table 1.

**Table 1:** Tissue processing protocol.

Step	Reagent	Duration
1	Water	1 h
2	Ethanol 70%	1 h
3	Ethanol 80%	1 h
4	Ethanol 96%	1 h
5	Ethanol 96%	1 h
6	Ethanol 100%	1 h
7	Ethanol 100%	1 h
8	Toluene	1 h
9	Toluene	1 h
10	Paraffin	2 h
11	Paraffin	2 h

Paraffin embedding (Fig. 2) was performed using triple-filtered paraffin with a melting point of 56–57 °C (Epredia, Histoplast PE 8330), using the equipment Arcadia H+C paraffin embedding station. Specimens were embedded flat, with the plane of the chorioallantoic membrane parallel to the cutting surface. This orientation was chosen so that each section would cover a large area of the graft and thus better represent the heterogeneous cell population expected for this cell line in the immunohistochemical evaluation. Embedding on edge, with the membrane perpendicular to the cutting surface, yields a higher proportion of sections containing both the graft and the underlying membrane and conserves tissue for subsequent analyzes, but each section then samples a smaller area of the graft and is correspondingly less representative for immunohistochemistry; a direct comparison of the two orientations is a natural extension of this protocol.

**Fig. 2:**
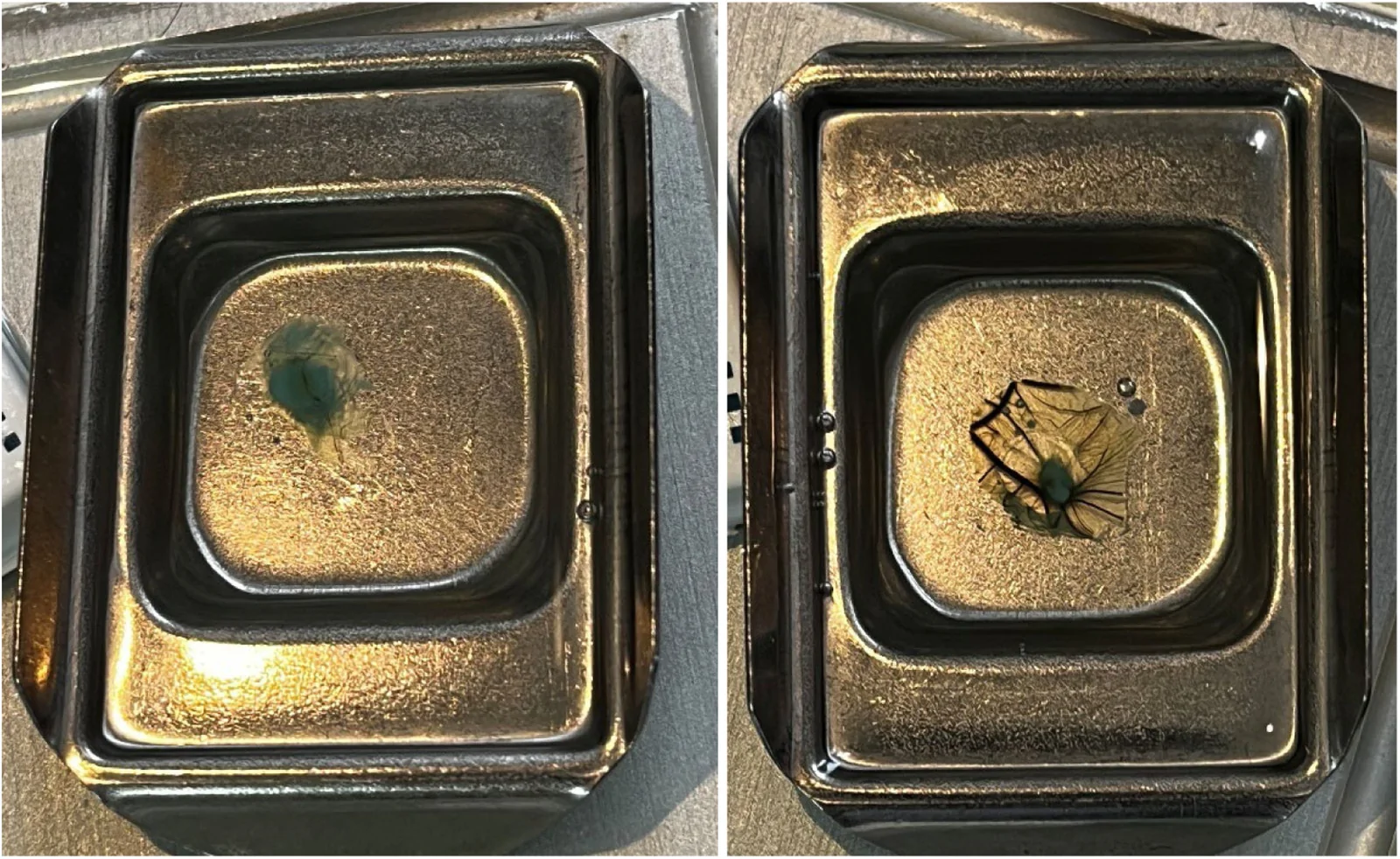
Two tumor specimens positioned in metal embedding molds, immersed in molten paraffin prior to solidification and tissue embedding.

Paraffin blocks were stored at 4 °C for 2 h prior to sectioning. Sections of 3 μm thickness were obtained using a microtome (Leica RM2245), which were then placed on plain glass slides for histology, and incubated for 1 h at 60 °C. Sections were subsequently stained with HE using an automated tissue stainer (Medite Tissue Stainer TCA-44), according to the protocol detailed in Table 2.

**Table 2:** Hematoxylin and eosin staining protocol.

Step	Reagent	Duration
**1**	Xylene	5 min
**2**	Xylene	5 min
**3**	Xylene	5 min
**4**	Ethanol 100%	1 min
**5**	Ethanol 100%	1 min
**6**	Ethanol 95%	1 min
**7**	Water	1 min
**8**	Hematoxylin	2 min
**9**	Water	1 min
**10**	Clarifier 1	30 s
**11**	Water	1 min
**12**	Bluing Reagent	1 min
**13**	Water	1 min
**14**	Ethanol 95%	1 min
**15**	Eosin Y	1 min
**16**	Ethanol 100%	1 min
**17**	Ethanol 100%	1 min
**18**	Ethanol 100%	1 min
**19**	Xylene	1 min
**20**	Xylene	1 min
**21**	Xylene	1 min

After HE staining, slides were examined under a brightfield microscope to assess tumor content, cellularity, and adequacy for IHC staining, which was subsequently performed.

For IHC, additional 3 μm sections were cut from the FFPE block showing the highest tumor cell content and adequate tissue preservation, as determined by review of the HE-stained slides by the study pathologist, and mounted on charged (silanized) slides. Staining was performed on a Leica BOND-MAX Fully Automated IHC Staining System, using the antibodies listed in Table 3, according to the protocols specified in the same table. Slides were subsequently mounted, scanned using a Leica Aperio AT2 scanner, and visualized digitally. Slides also included external positive controls, represented by human liver tissue, as well as the non-tumor-involved CAM as an internal control.

**Table 3:** Antibodies and protocols used for IHC staining.

Antibody	Clone	Manufacturer / Catalog no.	Protocol*	Epitope retrieval
**Anti-Human Hepatocyte**	OCH1E5	Agilent / GA624	RTU	HIER 20 min with ER2
**Multi-Cytokeratin**	AE1/AE3	Leica Biosystems / PA0909	RTU	Enzyme 1 for 10 min
**Arginase-1**	DBM15.12	Diagnostic BioSystems / PDM197	RTU	HIER 20 min with ER2

The protocol, including dilution, incubation time, and temperature for the primary antibody, followed the manufacturer's datasheet, as the antibodies used were in vitro diagnostic (IVD) reagents.

RTU: Ready-to-Use; HIER: Heat-Induced Epitope Retrieval; ER2: Epitope retrieval 2.

### Imaging and image analysis

The macroscopic documentation of the chorioallantoic membrane and the tumor graft was performed daily, from the moment of grafting until the day of collection (EDD14), using a Zeiss Discovery V8 stereomicroscope equipped with an Axiocam 105 camera. Images were captured for all experimental conditions (the three grafting intervals and both cell densities), including for the conditions in which grafting did not occur. Quantitative analysis of the images was performed using ImageJ/Fiji software (version 1.53). For each image, the scale was calibrated using the 500 µm scale bar present in the image (Analyze → Set Scale). The two-dimensional projected area of the tumor graft at the time of harvest (EDD14) was measured by manually outlining the opaque tumor mass with the freehand selection tool, followed by the measure command (Analyze → Measure). Measurements were performed on all 17 confirmed grafts obtained in the single condition that produced grafts (1 × 10⁶ cells grafted on EDD9; 17 of the 20 eggs allocated to that condition), and not on the full series of 120 grafted eggs; results are expressed as mean ± standard deviation. The area was reported as a two-dimensional projection of the grafted mass, without estimating the tumor volume. Measurements were performed by a single observer and inter-observer agreement was not assessed.

The images obtained daily between grafting and harvest were used exclusively for documentary purposes, without any morphometric or quantitative measurements; therefore, no graft growth curve was developed. In the unsuccessful experimental conditions, the area was not determined, as no well-defined mass was identified that would allow for a reproducible assessment.

### Statistical analysis

Graft formation was recorded for each of the 120 grafted eggs as a binary outcome (confirmed graft versus no confirmed graft), according to the criteria defined in Method details, and is reported as the observed proportion per condition with a 95% confidence interval calculated by the modified Wald method. Because the outcome was completely separated between conditions, with all confirmed grafts occurring in a single condition and none in the other five, no omnibus test across the six conditions was performed. Two pre-planned comparisons were analyzed with the two-tailed Fisher exact test: the successful condition against the same cell density applied on EDD7 and EDD8 taken together, and the successful condition against all five unsuccessful conditions taken together. The three replicate runs within the successful condition were compared pairwise with the same test. No correction for multiple comparisons was applied, and the pairwise analyzes are presented descriptively. Graft areas are summarized as mean ± standard deviation, with the 95% confidence interval based on the t distribution and the coefficient of variation. Embryo survival at EDD14 was compared between the two cell densities with the two-tailed Fisher exact test and between the three grafting days with the chi-square test. Because of the complete separation of the outcome, the p values obtained are extremely small and are reported only for completeness; the interpretation is based on the observed proportions and their confidence intervals. Analyzes and graphs were produced in GraphPad Prism version 11 and GraphPad QuickCalcs (GraphPad Software, Boston, MA, USA), with alpha set at 0.05, two-sided.

### Method validation

To establish the optimal grafting conditions, two cell densities were compared, namely 0.5 × 10⁶ and 1 × 10⁶ cells, applied at three distinct times of embryonic development: EDD7, EDD8 and EDD9. *The experiments were carried out as three replicate runs, each in a separate incubator.* All eggs were monitored until EDD14, with 20 eggs included for each experimental condition, corresponding to a total of 120 grafted eggs. The distribution of eggs between the three replicates, as well as the results obtained within each replicate, are presented in Table 4.

**Table 4:** Per-replicate outcome of HepG2 grafting onto the chorioallantoic membrane for each of the six tested conditions. Values are given as number of confirmed grafts / number of eggs grafted; the 20 eggs per condition were distributed as 7, 7 and 6 across the three replicate runs.

Cell density	Grafting day	Repeat 1	Repeat 2	Repeat 3	Total
**0.5** **×** **10⁶**	EDD7	0 / 7	0 / 7	0 / 6	0 / 20 (0%)
**0.5** **×** **10⁶**	EDD8	0 / 7	0 / 7	0 / 6	0 / 20 (0%)
**0.5** **×** **10⁶**	EDD9	0 / 7	0 / 7	0 / 6	0 / 20 (0%)
**1** **×** **10⁶**	EDD7	0 / 7	0 / 7	0 / 6	0 / 20 (0%)
**1** **×** **10⁶**	EDD8	0 / 7	0 / 7	0 / 6	0 / 20 (0%)
**1** **×** **10⁶**	EDD9	5 / 7	7 / 7	5 / 6	17 / 20 (85%)
**Total**					17 / 120

The allocation of eggs to the six conditions and across the three replicate runs is described in Method details and summarized in Supplementary Table S1.

A cohesive, well-vascularized mass that could be excised intact and processed appropriately for histological evaluation was obtained exclusively when a density of 1 × 10⁶ cells was used, grafted at EDD9. Under these conditions, confirmed grafts were obtained in 17 out of 20 eggs, corresponding to a proportion of 85% (95% CI: 62.9–95.4%, modified Wald method). Embryo survival was documented for all six conditions from the incubation records and the daily image series (Supplementary Table S1). Of the 120 grafted eggs, 100 embryos (83.3%) were alive at EDD14, the same number as at EDD12; survival per condition ranged from 15 of 20 to 18 of 20 (75–90%). Survival did not differ between the two cell densities (51/60 versus 49/60; two-tailed Fisher exact test, p = 0.81) or between the three grafting days (34/40, 31/40 and 35/40; chi-square test, p = 0.46), and was homogeneous across the six conditions (Fisher-Freeman-Halton exact test, p = 0.92). Differential embryo mortality therefore does not account for the difference in graft formation between conditions. In the condition that produced grafts, 17 of the 20 embryos were alive at EDD14 and 17 confirmed grafts were obtained; the records do not allow egg-by-egg matching of survival and graft confirmation, so these two counts are reported side by side without implying a one-to-one correspondence. All 17 grafts obtained in this condition were processed histologically and fulfilled the histological criterion defined in Method details.

Tumor graft formation occurred under only one experimental condition, whereas the other five conditions failed to yield any confirmed grafts. A direct comparison between the grafting of 1 × 10⁶ cells at EDD9 and the same cell density applied at EDD7 and EDD8 (analyzed collectively) revealed no confirmed grafts in the comparison group (0/40); furthermore, the two-tailed Fisher's exact test yielded a p-value of 2.9 × 10⁻¹². When compared against all five unsuccessful conditions analyzed collectively (0/100 confirmed grafts), the two-tailed Fisher's exact test yielded a p-value of 6.0 × 10⁻¹⁸.

Consequently, the primary interpretation relies on the observed proportions rather than the magnitude of the p-values.

The three replications of the successful experimental condition yielded consistent results, with 5/7, 7/7, and 5/6 confirmed grafts. Pairwise comparisons using two-tailed Fisher's exact tests revealed no significant differences between any pair of replicates (p = 0.46, p = 1.00, and p = 0.46), thereby confirming the reproducibility of the grafting condition based on the inoculation of 1 × 10⁶ HepG2 cells at EDD9.

At a density of 0.5 × 10⁶ cells, no cohesive mass was formed, regardless of the day on which the grafting was performed. Under conditions where 1 × 10⁶ cells were inoculated at EDD7 or EDD8, the presence of a whitish material inside the grafting ring was observed at EDD14. Under the EDD8 condition, this material sometimes showed a macroscopic extension apparently comparable to that observed in the successful condition (Fig. 3). However, the material did not show cohesiveness, disintegrated during harvesting, and could not be processed for histological evaluation. Consequently, it did not meet the predefined criteria for confirming the formation of a graft. Therefore, the macroscopic appearance alone is not a sufficient criterion for confirming grafting in this experimental model. The differentiation between experimental conditions is based on the cohesive character of the obtained material and the possibility of its sampling and histological processing, not just on the simple presence of an opaque material inside the graft ring.

**Fig. 3:**
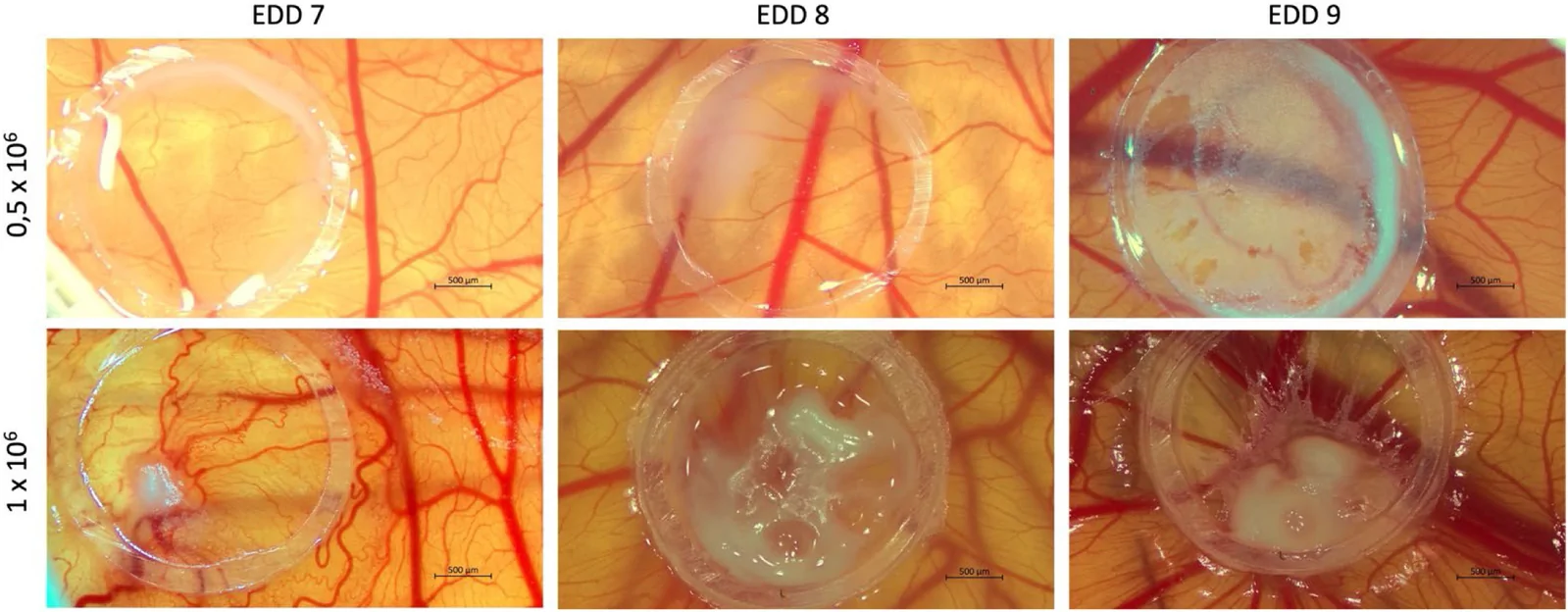
Optimization of HepG2 cell grafting onto the chorioallantoic membrane, depending on cell density (0.5 × 10⁶ vs. 1 × 10⁶) and grafting day (EDD7, EDD8, EDD9). Representative images acquired on EDD14 for each condition (n = 20 eggs/condition). Cohesive, vascularized, and histologically confirmable grafts were obtained exclusively with 1 × 10⁶ grafted cells on EDD9 (17/20; 85%). In other conditions, a confirmed graft was not formed, and the membrane remained viable; residual material observed in some cases was non-cohesive and disintegrated upon collection. Zeiss Discovery V8 stereomicroscope, Axiocam 105 camera. Scale bar: 500 µm.

The transition observed between EDD8 and EDD9 was abrupt and requires careful interpretation, given that the currently recommended grafting interval for the CAM model is generally between EDD8 and EDD10 [2,4]. During this period, the chorioallantoic membrane undergoes rapid changes in structural and vascular maturation: the capillary network develops intensively until around EDD8, and between EDD8 and EDD12, vascular remodeling processes through intussusceptive angiogenesis predominate [15]. Two non-exclusive explanations may be compatible with the observations obtained. First, the progressive thickening of the chorioallantoic membrane and the rapid reorganization of the capillary plexus during this interval may determine relevant differences between the available mesodermal surface at EDD8 and that at EDD9, with possible consequences on the establishment of an adequate interface between the inoculated cells and the membrane. The CAM becomes capable of supporting tissue grafts between EDD8–EDD10, and vascular differentiation continues dynamically during this developmental stage. Second, grafts applied at earlier time points remained on the membrane for a longer period before harvesting. In the absence of an extracellular matrix support such as Matrigel, it is possible that the deposited cells laterally dispersed across the expanding CAM surface rather than consolidating into a cohesive mass. This hypothesis is plausible in the context of the rapid growth and remodeling of the CAM between EDD8–EDD12, but cannot be directly demonstrated based on the current data set. Because harvesting was performed at a fixed time point, namely EDD14, the relative contribution of membrane maturity at the time of inoculation and that of the duration of post-grafting development cannot be separated in the present study. However, the fact that grafting was successful under the shortest development interval condition, inoculation at EDD9 followed by five days to harvest, while grafting at earlier times, associated with longer development intervals, did not lead to the formation of confirmed grafts suggests that the day of grafting is the predominant limiting parameter. This observation, however, does not allow establishing the biological mechanism responsible for the difference between EDD8 and EDD9.

The evolution of the HepG2 graft obtained by inoculating 1 × 10⁶ cells at EDD9 was documented daily by stereomicroscopy, from the moment of grafting until the harvest performed at EDD14. Fig. 4 illustrates the evolution of a representative graft, followed throughout the entire experimental interval.

**Fig. 4:**
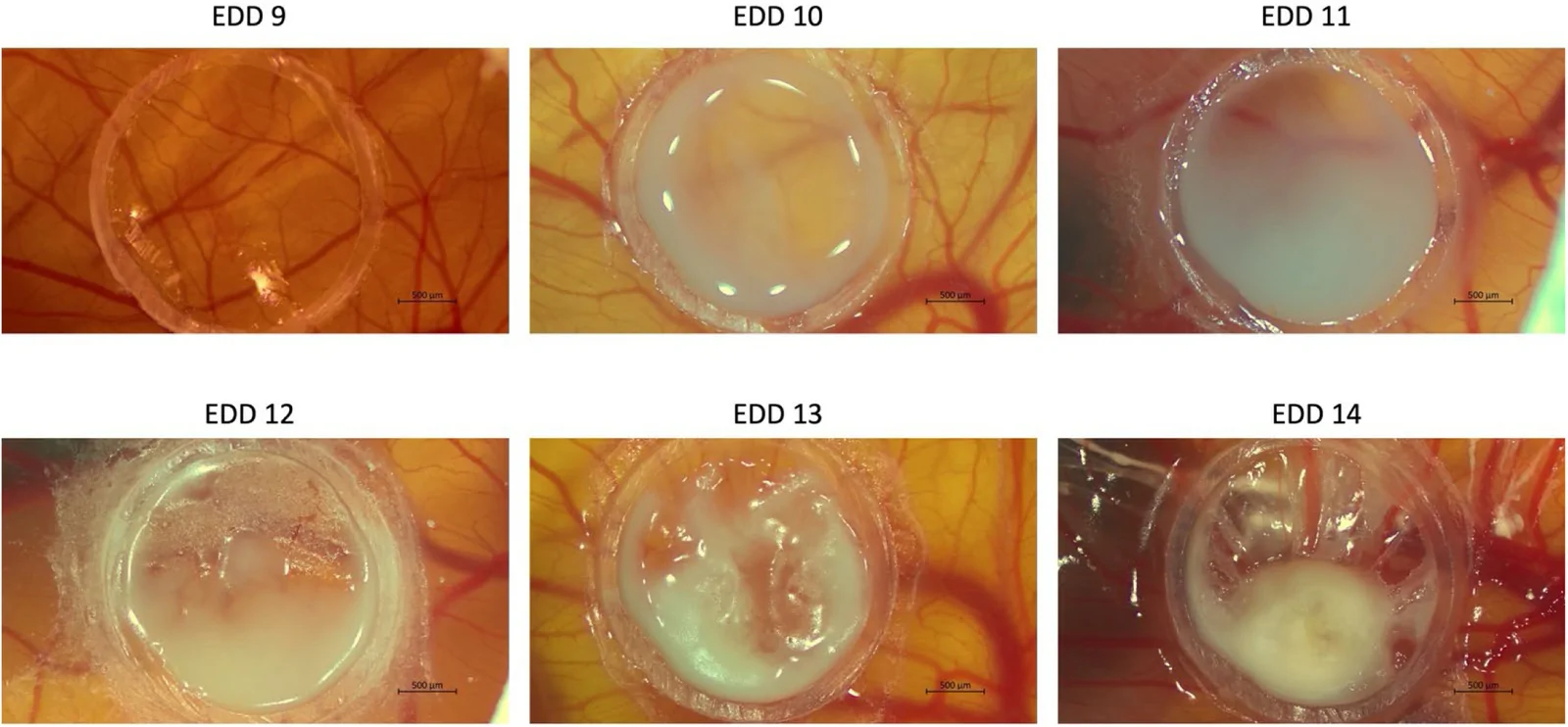
Daily stereomicroscopic documentation of one representative HepG2 graft (1 × 10⁶ cells, grafted on EDD9) on the chorioallantoic membrane, from grafting (EDD9) until harvest (EDD14); the same egg is shown on each day. An opaque mass is visible inside the polyethylene ring from EDD10 onwards, the early opacity including the deposited cell suspension, and becomes progressively more compact and better delimited; CAM vessels converge toward the periphery of the graft. No area measurements were performed on the intermediate days. Zeiss Discovery V8 stereomicroscope, Axiocam 105 camera. Scale bar: 500 µm.

Immediately after the application of the cell suspension, at EDD9, it could not be macroscopically delimited, and the polyethylene ring circumscribed a well-vascularized area of ​​the chorioallantoic membrane. Starting with EDD10, an opaque, whitish mass became visible inside the ring. However, part of this early opacity probably corresponds to the initially deposited cell suspension; therefore, the images obtained at EDD10 and EDD11 should not be interpreted as directly indicating tumor growth.

During the interval EDD11–EDD13, the mass became progressively more compact and better delimited. At the time of harvest, at EDD14, it presented the appearance of a cohesive mass, with a clear outline, opaque white color and organized peripheral vascularization. The blood vessels in the vicinity presented a radial distribution, “spoke wheel” type, oriented toward the periphery of the grafted mass (Fig. 4).

Since the projected area was measured only at the time of harvest, the daily images had an exclusively documentary role and do not constitute a quantitative growth curve. Thus, the persistence of a cohesive and well-demarcated mass at EDD14 is reported as a descriptive observation, not as a kinetic growth parameter.

Under these experimental conditions, engraftment was reproducible in 17 out of 20 eggs, corresponding to a confirmation rate of 85% (95% CI 62.9–95.4%). The obtained mass could be excised intact and subsequently processed for histological evaluation.

To provide a dimensional characterization of the graft at the time of harvest, the tumor area was measured on stereomicroscopic images acquired at EDD14 using ImageJ/Fiji (version 1.53) [16,17]. Areas were quantified for all 17 successful grafts; the individual values are listed in Table 5. For this purpose, the scale was calibrated using the 500 µm bar present in each image. The mean projected graft area was 1.52 ± 0.28 mm² (95% CI 1.37–1.66 mm²; range 1.12–2.10 mm²; coefficient of variation 18.5%; n = 17). Measurements were performed by a single observer; inter-observer agreement was not assessed. This value represents a two-dimensional projection of the graft mass and not a volumetric estimate (Fig. 5).

**Table 5:** Individual projected graft areas measured on stereomicroscopic images at EDD14 for the optimal condition (1 × 10⁶ cells grafted on EDD9). Grafts are identified by sequential egg identifier, not by area. All 17 confirmed grafts were measured.

Graft	Projected area at EDD14 (mm²)
**G01**	2.10
**G02**	1.98
**G03**	1.65
**G04**	1.55
**G05**	1.45
**G06**	1.32
**G07**	1.23
**G08**	1.42
**G09**	1.33
**G10**	1.72
**G11**	1.28
**G12**	1.12
**G13**	1.39
**G14**	1.27
**G15**	1.37
**G16**	1.74
**G17**	1.85
**Mean ± SD (n** **=** **17)**	1.52 ± 0.28
**95% CI**	1.37 – 1.66

**Fig. 5:**
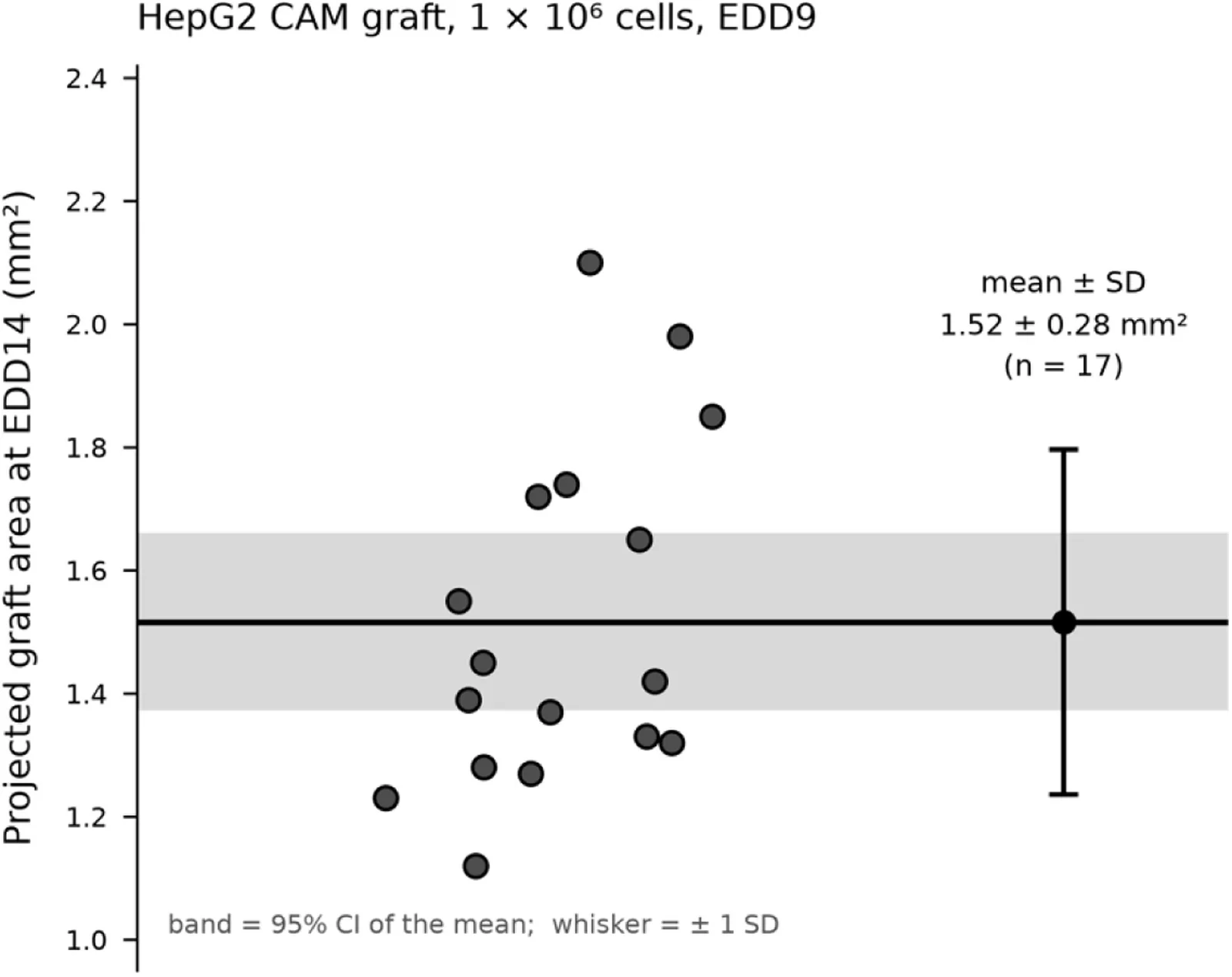
2D projected area of the HepG2 graft at the time of collection (EDD14), for the optimal grafting condition (1 × 10⁶ cells, EDD9). Each point represents an individual graft (n = 17); the horizontal line indicates the mean (1.52 mm²), the shaded band the 95% confidence interval of the mean (1.37–1.66 mm²), and the whisker ± 1 standard deviation (± 0.28 mm²). The area was measured in ImageJ/Fiji, with scale calibration based on the 500 µm bar. Values represent two-dimensional projections, not volumetric estimates.

On histological examination by hematoxylin–eosin (HE) staining (Fig. 6A–C), the graft was represented by a cellular mass located at the level of the chorioallantoic membrane (Fig. 6A). In some sections, central areas showed the tumor in contact with portions of CAM mesoderm (Fig. 6B). The tumor was composed of a diffuse proliferation of atypical cells, arranged predominantly in a solid pattern, with focal trabecular arrangement. The cells showed an increased nuclear/cytoplasmic ratio, eosinophilic cytoplasm, and nuclear atypia, including hyperchromatic nuclei, mildly irregular nuclear membranes, and focal multinucleation. Atypical mitotic figures were identified as well (Fig. 6C). Overall, the morphological aspect was consistent with a malignant tumor.

**Fig. 6:**
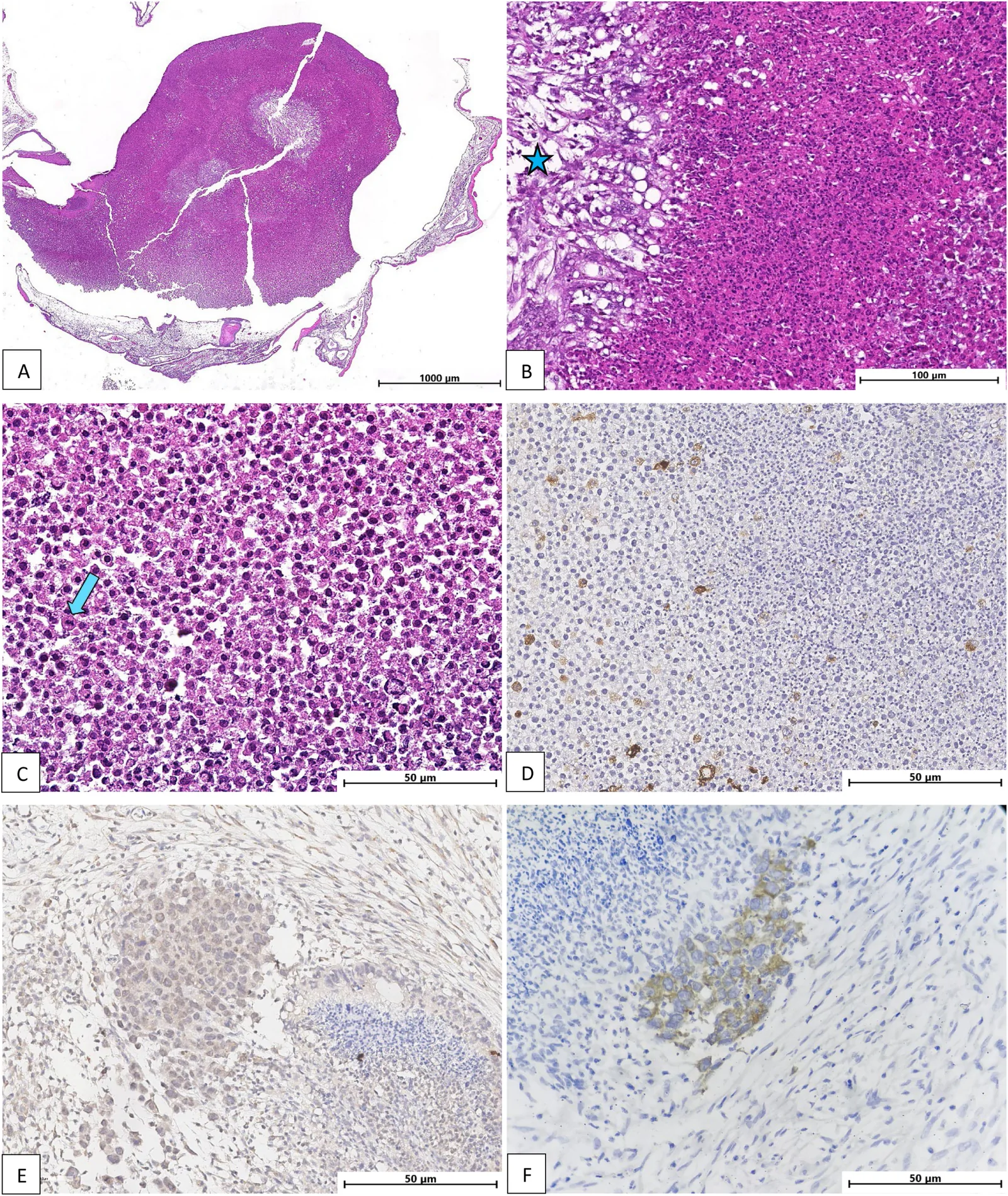
Histopathological and immunohistochemical features of the CAM-engrafted tumor. (A) Low-power view of the developed tumor, corresponding to the gross appearance, with parts of the chorioallantoic membrane visible (HE stain). (B) Interface between the tumor and the CAM, showing CAM mesoderm (pointed by the star) on the left and tumor on the right (HE stain). (C) High-power view showing tumoral cells and an atypical mitotic figure pointed by the blue arrow. (HE stain). (D) Tumoral cells with faint positivity for pancytokeratin (AE1/AE3). (E) Faint granular cytoplasmic and nuclear staining for Arginase-1. (F) Tumoral cells showing cytoplasmic positivity for HepPar-1 (hepatocyte-specific antigen). Scale bars: 1000 µm (A), 100 µm (B), 50 µm (C, D, E, F).

Immunohistochemical analysis was performed to further characterize the graft. Arginase-1, HepPar-1 and pan-cytokeratin marker AE1/AE3 showed focal staining in different components of the tumor (Fig. 6D–F). Pan-cytokeratin AE1/AE3 is expressed in the majority of epithelial tumors, including hepatic ones, albeit often only focally [18]. Arginase-1 and HepPar-1 are important markers of hepatocellular differentiation, with sensitivity decreasing significantly in poorly differentiated tumors [19]. Thus, the focal, but relatively moderate to intense reactivity observed in the analyzed grafts is consistent with the profile of these markers in poorly differentiated hepatocellular malignant tumors [20].

This immunohistochemical profile suggests a malignant tumor of hepatocellular origin, with the patchy staining pattern for these markers being more indicative of hepatoblastoma than hepatocellular carcinoma. Confirmation of the origin and tumor subtype would require molecular analysis of the grafted material; however, this could not be performed in our case, as insufficient tissue remained following haematoxylin–eosin and immunohistochemical analysis. Embedding on edge would conserve tissue for such analyzes in future applications of the protocol.

## Conclusions

This work was undertaken because the chorioallantoic membrane assay is widely used as a rapid, inexpensive and ethically favorable alternative to rodent xenografts, yet no systematic comparison of grafting conditions was available for HepG2, the most widely used human hepatic tumor cell line. Published CAM protocols for hepatic cells specify a cell number and a grafting day without reporting how either parameter was selected, which leaves laboratories to re-optimize the assay empirically.

Two findings emerged. First, cell density and grafting day are not independent, gradually acting variables in this model: within a single standardized framework, a cohesive and histologically processable graft was obtained only when both were set correctly, with 1 × 10⁶ cells inoculated on EDD9 (17 of 20 eggs, 85%), whereas all five remaining combinations yielded no processable graft (0 of 100). Second, the transition between EDD8 and EDD9 was abrupt rather than progressive, which indicates that the grafting day is the limiting parameter within the window usually recommended for the CAM assay, and that a shift of a single day can determine whether the assay succeeds or fails.

Relative to existing approaches, the protocol offers three practical advantages. It is fully matrix-free and uses a 5 µL inoculum inside a laboratory-made polyethylene ring, so that it can be reproduced at minimal cost and with a defined composition, without the lot-to-lot variability introduced by basement membrane extracts. It produces, within five days, a graft that can be excised intact and processed for histology and immunohistochemistry, so that the morphological readout is available in the same experimental run. And, because the five unsuccessful combinations are reported in full rather than omitted, it provides laboratories setting up the assay with a map of the conditions that do not work, which is normally the most time-consuming part of establishing a CAM model. All procedures are completed by EDD14, before the stage at which the avian embryo is afforded regulatory protection, so the method remains consistent with the 3Rs.

## Limitations

The method described earlier has some limitations that are worth highlighting. First, the harvest day, EDD14, was the same for all the experiments conducted. Thus, the growth duration of the graft varied between conditions, being 7, 6, and 5 days for grafts performed on EDD7, EDD8, and EDD9, respectively. However, successful grafting was obtained under the condition with the shortest growth interval (EDD9, 5 days), suggesting that the timing of grafting, rather than the duration of growth, is the predominant factor, although it is not possible to separate the two parameters fully in this study.

Second, in the experimental conditions in which a cohesive graft was not obtained, the residual material observed stereomicroscopically lacked cohesiveness and disintegrated during harvesting. This situation prevented histological processing of the material and, implicitly, confirmation of its nature.

In the condition characterized by inoculation of 1 × 10⁶ cells at EDD8, the residual material sometimes presented a macroscopic appearance comparable to that observed in the successful condition. Therefore, the differentiation between these two conditions is supported at the level of cohesiveness and the possibility of sampling and histological processing of the material, not at the level of tissue composition, which could not be evaluated in the unsuccessful conditions.

A third limitation concerns the characterization of the graft, as the human origin and subtype of the grafted mass was not confirmed molecularly. Short tandem repeat authentication of the cell line [21], human-specific Alu qPCR [22] or mitochondrial DNA digital PCR [23] on the graft, and molecular testing for the cell-line-specific mutations reported by the distributor, were not performed; the insufficient amount of tumoral tissue available precluded these analyzes at this stage. Part of this limitation follows from the flat embedding orientation chosen here; embedding on edge would conserve tissue and leave material available for molecular confirmation, at the cost of a smaller tumor area per section. Consequently, although the histological appearance and weak immunohistochemical reactivity for HepPar-1 and Arginase-1 are compatible with the presence of a grafted HepG2 cell population, the available data do not allow for the formal exclusion of the possibility that some of the mass observed within the ring corresponds to a host reaction. Closely related to this limitation, the experimental design did not include a vehicle control condition, namely the application of a ring containing 5 µL of culture medium, associated with an identical membrane lesion, but in the absence of cells. Such a control is necessary to determine to what extent the material observed inside the ring can be attributed to inoculated cells, compared to the response induced by mechanical damage to the CAM and the presence of the polyethylene ring. This control should be included and investigated in parallel by laboratories implementing the protocol. Its absence is the main reason why the histological and immunohistochemical results are presented in this study with exclusively descriptive value. Furthermore, the membrane's reaction to the polyethylene ring and the micro-injury was expected to manifest to a comparable degree across all six experimental conditions, given that the ring was applied in an identical manner. However, a cohesive mass suitable for histological processing was obtained in only one of these conditions. This finding argues against a purely reactive origin of the graft, yet it does not eliminate the need for a vehicle control group. We recommend this measure to laboratories implementing the protocol. A further limitation concerns the deliberate omission of an exogenous extracellular matrix. A Matrigel-supported arm was not tested in parallel, and we therefore cannot state whether a matrix would have raised the 85% confirmation rate obtained in the optimal condition, or whether it would have permitted engraftment at the lower cell density or at the earlier grafting days. Matrix support can be clearly advantageous in other CAM models. A matrix-supported variant is consequently the first extension we recommend to laboratories adopting this protocol, in particular for cell lines with weaker intrinsic adhesion. It should be noted that matrix-supported inocula and cell numbers above 1 × 10⁶ require a considerably larger application volume, which is not compatible with the 5 µL drop and the 5 mm ring used here; the ring size and the application volume would therefore have to be adapted accordingly. For the same reason, cell densities above 1 × 10⁶ cells per egg were not evaluated; testing them would require a larger inoculation volume and a correspondingly larger ring, and this, together with a matrix-supported arm, constitutes a natural extension of the present protocol.

An additional limitation is of a technical nature and concerns the euthanasia step. Embryos were euthanized by hypothermia, by maintaining at −20 °C for 5 h, prior to in situ fixation. Although the use of cooling below 4 °C or freezing is accepted for avian embryos <80% of the incubation period [14], this approach exposes the tissue to freezing temperatures before it is stabilized by chemical fixation. It is also slower than decapitation or an anesthetic overdose. For both reasons, an anesthetic overdose administered before fixation is the refinement we recommend to laboratories adopting the protocol [9]. Immunohistochemistry was performed on specimens fixed for approximately 48 h in total, using the retrieval conditions validated for diagnostic specimens in our laboratory. Although this duration lies within the range routinely applied for immunohistochemistry, no parallel series fixed for 24 h was processed, and a contribution of fixation time to the intensity of staining cannot be formally excluded.

The main evaluation criterion in the optimization process was a binary assessment by the operator, namely the presence or absence of a cohesive graft suitable for histological processing, applied according to the criteria defined in the Method details section. The operator was not blinded to the experimental condition, and the assessment was not verified by a second independent reading; therefore, a certain degree of subjectivity in the classification of the results cannot be excluded. Because fragmentation during harvesting leads to classification as an unconfirmed graft regardless of the histological nature of the material, the reported engraftment rate is a lower bound.

For future comparisons, it is recommended to use quantitative evaluation criteria, less dependent on the operator's judgment, such as the wet weight of the excised fragment or the projected area, systematically measured in all experimental conditions.

Embryonic viability was not scored prospectively on the intermediate days of incubation. Candling on EDD3 and immediately before grafting documents the number of viable embryos at the start, and survival at EDD12 and EDD14 could be established retrospectively from the incubation records and the daily image series (Supplementary Table S1); the exact day of death of the 20 embryos that were lost cannot, however, be determined, and no intermediate viability curve can be derived. Nor do the records permit egg-by-egg matching between embryo survival and graft confirmation. These residual gaps limit the interpretation of the losses, but they do not affect the comparison between conditions, since survival at EDD14 was homogeneous across all six. We recommend that any subsequent use of this protocol record prospectively, and report, the complete experimental flow: the number of eggs set for incubation, the number of fertile eggs at candling, the number of eggs successfully windowed, the number of eggs grafted, the identity of each egg, and the viability of each embryo at every daily observation until harvest. Because the definition of the denominator determines every reported proportion, this level of reporting is essential for comparability between laboratories. Also, all experiments were performed in the in ovo system. Since the in ovo and ex ovo variants of the CAM assay differ in terms of chorioallantoic membrane maturation, angiogenesis processes and tumor development dynamics, the conditions defined in the present study should not be directly transferred to ex ovo cultures without prior experimental revalidation [24]. Two features of the egg supply limit the generality of the present results. The eggs were obtained from a small, non-certified local family farm and came from a mixed-breed layer flock of undefined genetic line, rather than from a defined commercial line supplied by a registered hatchery; and, although the three replicate runs were performed in separate incubators, they all used eggs from a single batch, which was a deliberate choice to exclude batch-related variation from the comparison between conditions but which means that reproducibility across independent egg batches and flocks has not been established. Laboratories adopting the protocol should preferably use eggs of a defined commercial line and, where possible, replicate across batches.

In the end, the model is limited to the harvest day EDD14, set in accordance with ethical considerations regarding the use of the model in ovo (as a precautionary measure related to the possible onset of nociception). This limited time window greatly restricts any subsequent experimental interventions on the graft.

## Ethics statements

CAM (chorioallantoic membrane) had been used as a model of chick embryo chorioallantoic membrane in ovo in this study. A local commercial supplier provided fertilized chicken eggs (gallus domesticus). According to Article 1, paragraph 3 of Directive 2010/63/EU on the protection of animals used for scientific purposes, as well as Law No 43/2014, which transposes that Directive into Romanian law, pre-hatch avian embryos are not protected animals. In light of this, the described procedures do not fall under the jurisdiction of regulations governing animal experimentation, and approval from an animal ethics committee was not required [7,8]. As a precautionary welfare measure, all experiments were terminated on embryonic development day 14 (EDD14), before hatching and before the final third of the 21-day incubation period [9]. The embryos were euthanized on EDD14 via hypothermia (−20 °C for 5 h) prior to fixation and tissue harvesting, in accordance with the AVMA Guidelines for the Euthanasia of Animals [14]. Accordingly, the study was designed and reported in accordance with the principles of the 3Rs, as well as the guidelines of ARRIVE 2.0, to the extent that they apply to an in ovo model. The sex of the embryos was not determined, as it cannot be established at this developmental stage without molecular analysis. There was no involvement of privately owned animals or threatened or endangered species in this study.

## CRediT authorship contribution statement

**Cristina Adriana Dehelean**: Conceptualization, Data curation, Writing-Original draft preparation. **Diana Haj Ali**: Methodology, Data curation. **Robert Barna**: Investigation, Methodology, Writing-Reviewing. **Iasmina Predescu**: Methodology, Software. **Ioana Macasoi**: Conceptualization, Validation, Supervision, Writing-Reviewing, Editing.

## Funding

This research did not receive any specific grant from funding agencies in the public, commercial, or not-for-profit sectors.

## Declaration of generative AI and AI-assisted technologies in the manuscript preparation process

During the preparation of this work the authors used Claude (Anthropic) in order to check the formatting and consistency of the reference list and in-text citations, identify potentially relevant literature, improve the language and readability of the text, and obtain critical feedback on the clarity and completeness of the manuscript. After using this tool, the authors reviewed and edited the content as needed, verified all references against the original sources, and take full responsibility for the content of the published article.

## Declaration of competing interest

The authors declare that they have no known competing financial interests or personal relationships that could have appeared to influence the work reported in this paper.

## Data Availability

Data will be made available on request.
