# Supplementary material for: Influence of cell seeding density and inoculation timing on HepG2 cell engraftment on the chick chorioallantoic membrane
Source: MethodsX. 2026 Nov 3;17:104241. doi: 10.1016/j.mex.2026.104241 (PMC13769227; doi:10.1016/j.mex.2026.104241)
Supplement: Supplementary file 1 [file mmc1.docx]

**Supplementary Table S1.** Flow of eggs through the study and embryo survival by experimental condition.

**Part A. Flow of eggs**

*All eggs originated from a single batch and were incubated simultaneously in three incubators, one per replicate run.*

| **Stage** | **Number of eggs** | **% of eggs set** |
| --- | --- | --- |
| Eggs set for incubation | 160 | 100 |
| Viable embryos at candling (EDD3) | 147 | 91.9 |
| Successfully windowed (EDD4) | 140 | 87.5 |
| Allocated to the six conditions and grafted | 120 | 75.0 |
| Kept as reserve, not grafted | 20 | 12.5 |
| Embryos alive at EDD12 | 100 | — |
| Embryos alive at EDD14 (harvest) | 100 | — |

**Part B. Embryo survival and graft formation by experimental condition**

| **Cell density** | **Grafting day** | **Eggs grafted** | **Embryos alive at EDD14 (%)** | **Confirmed grafts (%)** |
| --- | --- | --- | --- | --- |
| 0.5 × 10⁶ | EDD7 | 20 | 17 (85) | 0 (0) |
| 0.5 × 10⁶ | EDD8 | 20 | 16 (80) | 0 (0) |
| 0.5 × 10⁶ | EDD9 | 20 | 18 (90) | 0 (0) |
| 1 × 10⁶ | EDD7 | 20 | 17 (85) | 0 (0) |
| 1 × 10⁶ | EDD8 | 20 | 15 (75) | 0 (0) |
| 1 × 10⁶ | EDD9 | 20 | 17 (85) | 17 (85) |
| **Total** | **—** | **120** | **100 (83.3)** | **17 (14.2)** |

Percentages for embryo survival and for confirmed grafts are calculated on the number of eggs grafted in each condition. No further embryo losses occurred between EDD12 and EDD14. Embryo survival did not differ between the two cell densities (51/60 versus 49/60; two-tailed Fisher exact test, p = 0.81) or between the three grafting days (34/40, 31/40 and 35/40; chi-square test, p = 0.46), and was homogeneous across the six conditions (Fisher–Freeman–Halton exact test, p = 0.92). A confirmed graft was defined as a cohesive mass that met the criteria specified in Method details. EDD, embryonic development day.
